# Pitcher geometry facilitates extrinsically powered ‘springboard trapping' in carnivorous *Nepenthes gracilis* pitcher plants

**DOI:** 10.1098/rsbl.2022.0106

**Published:** 2022-08-03

**Authors:** Anne-Kristin Lenz, Ulrike Bauer

**Affiliations:** School of Biological Sciences, University of Bristol, 24 Tyndall Avenue, Bristol BS8 1TQ, UK

**Keywords:** carnivorous plant, trapping mechanism, plant movement, biomechanics, computed micro-tomography

## Abstract

Carnivorous pitcher plants capture insects in cup-shaped leaves that function as motionless pitfall traps. *Nepenthes gracilis* evolved a unique ‘springboard' trapping mechanism that exploits the impact energy of falling raindrops to actuate a fast pivoting motion of the canopy-like pitcher lid. We superimposed multiple computed micro-tomography images of the same pitcher to reveal distinct deformation patterns in lid-trapping *N. gracilis* and closely related pitfall-trapping *N. rafflesiana*. We found prominent differences between downward and upward lid displacement in *N. gracilis* only. Downward displacement was characterized by bending in two distinct deformation zones whist upward displacement was accomplished by evenly distributed straightening of the entire upper rear section of the pitcher. This suggests an anisotropic impact response, which may help to maximize initial jerk forces for prey capture, as well as the subsequent damping of the oscillation. Our results point to a key role of pitcher geometry for effective ‘springboard' trapping in *N. gracilis*.

## 1. Introduction

Some of the fastest plant movements are used by carnivorous plants to capture their agile prey [1]. In contrast to animals, plants generate movement without muscles. Instead, they employ growth or water transport processes to accumulate elastic energy, which is suddenly released via snap-buckling or explosive fracture [2]. Whist these spring mechanisms generate astonishingly fast movements [3], they require time and metabolic energy for preloading [4]. For a carnivorous plant, this could mean missing a catch because the trap is not ready. The pitcher plant *Nepenthes gracilis* solved this problem elegantly: it exploits falling rain drops to generate a fast and instantaneous trap movement free of metabolic costs [4,5].

**Figure 1.**
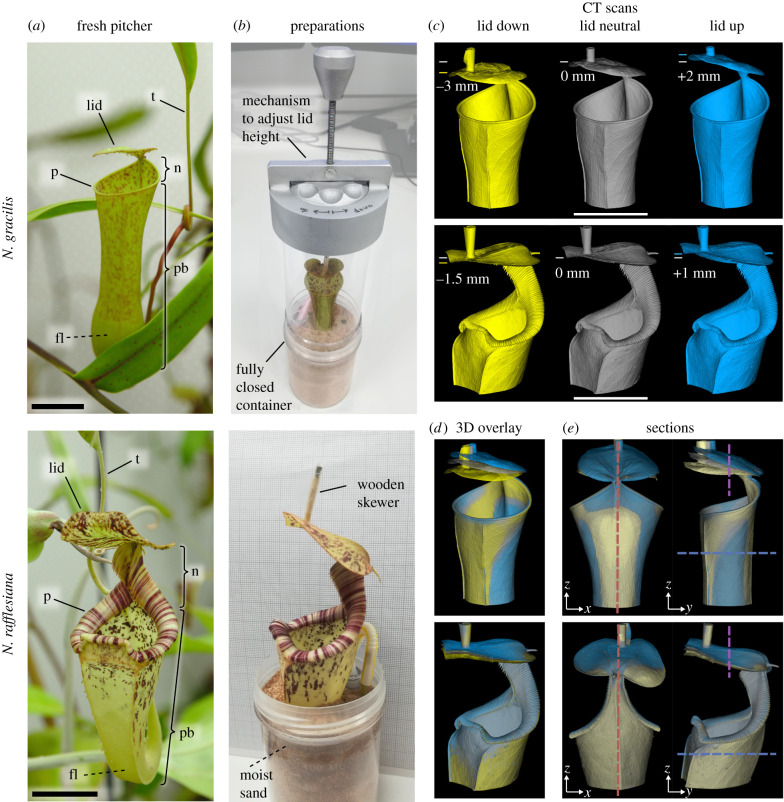
Experimental procedure to visualize pitcher deformation associated with lid displacement (scale bars = 25 mm). (*a*) Pitchers of *N. gracilis* (top) and *N. rafflesiana* (bottom); t, tendril; p, peristome; n, neck; pb, pitcher body; fl, pitcher fluid. (*b*) For µ-CT scanning, the pitcher was embedded in moist sand within a sealed container. The position of the pitcher lid could be adjusted via a device in the container lid. (*c*) Each pitcher was scanned with three different lid positions: neutral (grey), downward (yellow) and upward (blue), and (*d*) the resulting three-dimensional models were overlaid. (*e*) Longitudinal sections through the dorsal spine (red) and cross-sections through the pitcher body and neck (blue) and through the lid (purple) were used to analyze the deformation.

Conventionally, pitcher plants capture prey with motionless pitfall traps (figure 1*a*). Insects are attracted by nectar on the trap rim (peristome) and underneath the roof-like pitcher lid [6,7]. Slippery surfaces on the peristome [8] and inner pitcher wall [9,10] cause prey to fall into the fluid-filled trap where they drown and are digested. The lid prevents dilution of the pitcher contents with rainwater [11] and is not normally involved in trapping. *Nepenthes gracilis* uniquely uses a ‘springboard' action of the lid to capture prey. Impacting rain drops elicit a rapid oscillation that catapults insects into the trap. This additional trapping mechanism significantly increases prey intake in the field [5] and relies on three crucial adaptations: first, an approximately horizontal lid, so that insects are accelerated into the trap. Second, a lower lid surface which insects can access while the lid is still, but fall off during movement. Finally, a stiff lid for transmitting the impact force to the prey, attached to an elastic structure that facilitates the ‘springboard' action [12].

Previous comparisons of *N. gracilis* and sympatric, pitfall-trapping *Nepenthes rafflesiana* established that the stiff-plate pivoting motion of the *N. gracilis* lid produces high jerk forces over almost the entire area of the lower lid surface, and postulated the existence of a ‘torsion spring' in the ‘neck' region between pitcher body and lid [12]. Here, we apply serial computed micro-tomography (µ-CT) scanning to investigate the impact response of *N. gracilis* pitchers in detail. We hypothesize that the characteristic impact response is facilitated by elastic deformations that are spatially separated from the lid.

## 2. Material and methods

Plants were kept in a climate-controlled growth chamber simulating the natural growth conditions (electronic supplementary material: Methods). Mature aerial pitchers [6] of *N. gracilis* (*n* = 6) and *N. rafflesiana* (*n* = 5), each from a different plant, were harvested at least one week after opening. Each pitcher was rinsed with water to remove contamination, embedded in a container with sand up to approximately one-third of the pitcher height (figure 1*b*), and fixed in position by moistening the sand. Using fast drying epoxy glue (Wilko Ltd, Worksop, UK), a wooden skewer was attached to the upper lid surface, approximately three quarters along the proximal-distal center line. The skewer was connected to a custom-built device in the container lid (figure 1*b*) which enabled the position of the lid to be adjusted with an accuracy of 0.5 mm.

**Figure 2.**
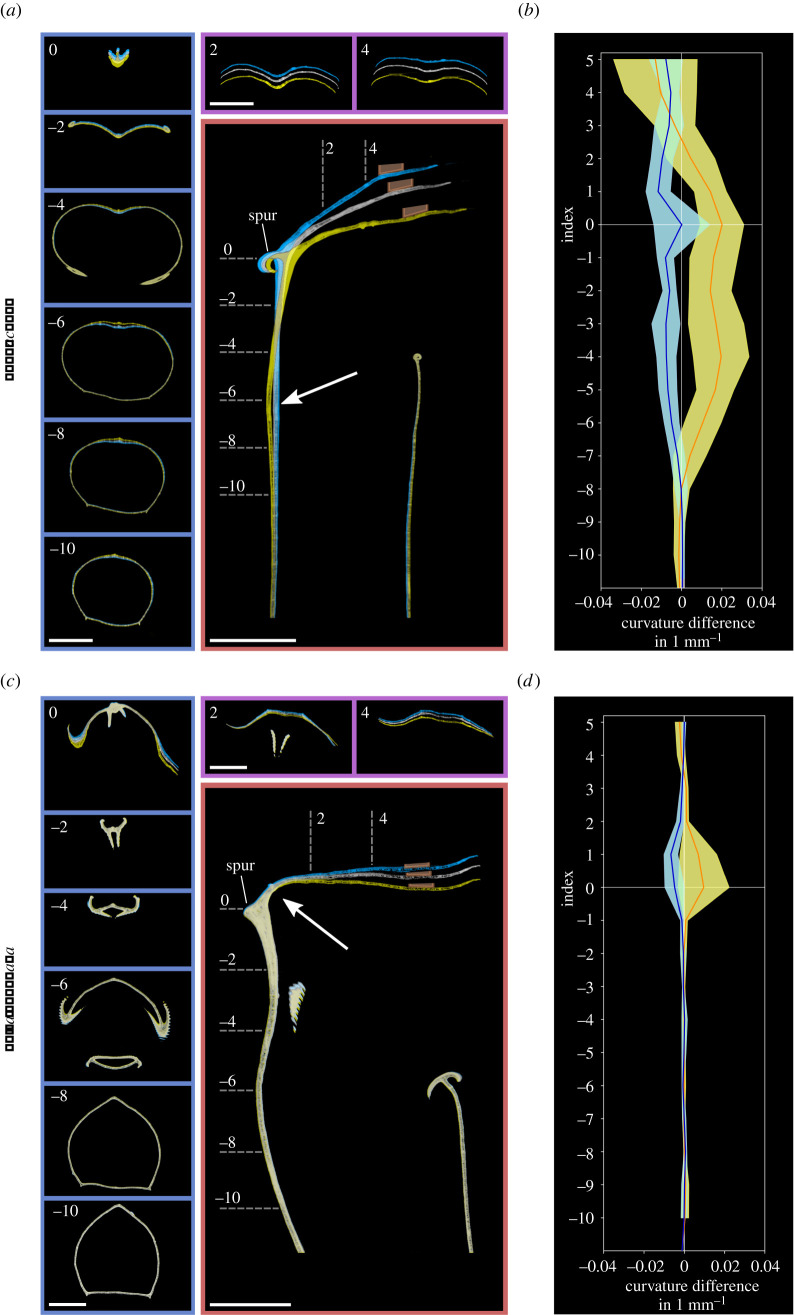
Pitcher deformation patterns for *N. gracilis* (*a*,*b*) and *N. rafflesiana* (*c*,*d*) visualized from overlays of µ-CT scans with three different lid positions (neutral, grey; downward, yellow; upward, blue). Whilst *N. gracilis* deforms mostly along the dorsal spine of the upper pitcher body, *N. rafflesiana* deforms in the basal part of the lid. (*a*,*c*) Overlaid longitudinal (red frame) and cross-sections (blue and purple frames) of the upper pitcher body and lid (scale bars = 10 mm). Arrows point to the areas of strongest deformation. The position of the manipulating skewer is indicated in the longitudinal sections. Indices mark the locations of the individual cross-sections. (*b*,*d*) Differences in the curvature of the dorsal pitcher spine between neutral and downward (orange) and upward (blue) lid positions, determined from the longitudinal sections of six *N. gracilis* (*b*) and five *N. rafflesiana* (*d*) pitchers. Lines denote means and shaded areas indicate 95% confidence intervals.

Each pitcher was imaged in a Nikon XT H 225 ST µ-CT scanner (XTM Facility, Palaeobiology Research Group, University of Bristol, for details see electronic supplementary material: Methods) three times with different lid positions in randomized order. Lid positions resembled the extreme upward and downward positions during an impact-induced oscillation (averaged from high-speed videos [12]), and the neutral position of the undisturbed lid (determined from photographs). Because *N. gracilis* lids respond to drop impacts with larger displacement than those of *N. rafflesiana* [12], the applied displacement differed between the species (figure 1*c*). From the scans, three-dimensional models were reconstructed and superimposed (Figure 1*d*). Longitudinal two-dimensional sections (figure 1*e*) in the bilateral symmetry plane were overlaid to show deformation along the dorsal spine, i.e. the rear quadrant of the pitcher tube and its continuation into the lid (figure 2*a,c*). Cross-sections were taken horizontally through the tubular pitcher body and the pitcher neck, and vertically through the lid (figure 1*e*). The dorsal spur (figure 2*a,c*) defined the boundary between neck and lid.

Equidistant points were fitted along the dorsal spine for each lid position, from the lowest point in the pitcher body to the attachment point of the skewer. Based on a convergence study and the visible height of each pitcher (electronic supplementary material: Methods and figure S1), six points (positive indices) were placed in the lid, and between seven and 26 points (negative indices) along the spine of the pitcher body. The curvature *κ* in each point was calculated as

2.1 $$\kappa =\frac{y^{\prime }z^{\prime \prime }-z^{\prime }y^{\prime \prime }}{{\left(y^{{\;}^{\prime }2}+z^{{\;}^{\prime }2}\right)}^{1.5}},$$

where *y* and *z* are coordinates of each point along the spine. From this, local changes in spine curvature between the three different lid positions were calculated (figure 2*b,d*).

To estimate the resistance to lid displacement in both directions, we loaded 10 *N. gracilis* lids by attaching two magnets (total mass = 3.5 g) in the same location as the skewer in our scans, resulting in a similar lid displacement as in the CT scans. Each pitcher was photographed with and without load, in upright and inverted orientation in randomized order, and next to a ruler for scale, using a digital SLR camera (Canon Inc., Tokyo) with 90 mm macro lens. Corresponding images with and without load were overlaid, and the displacement of the distal lid tip was measured in triplicate.

## 3. Results

The µ-CT images revealed clearly distinct deformation patterns for *N. gracilis* and *N. rafflesiana* (figure 2). We found prominent differences between downward and upward lid displacement in *N. gracilis* only (figure 2*a,b*), where downward lid displacement was characterized by deformation of the pitcher body and neck, away from the lid (figure 2*a*). By contrast, downward and upward lid movement in *N. rafflesiana* was effected by bending in the proximal quarter of the lid itself (indices 0 to +1; figure 2*c,d*).

In *N. gracilis,* the deformation was mainly confined to the dorsal side of the pitcher. During downward lid displacement, all *N. gracilis* pitchers showed an increase in curvature (bending) in two distinct areas of the dorsal spine (figure 2*b*): (i) the neck between pitcher body and lid (index 0), and (ii) the tubular pitcher body (indices −8 to −3). The interjacent region resisted deformation. The exact position of the lower deformation zone varied between pitchers; however, it was invariably associated with a transition of the cross-section from circular in the lower, to kidney-shaped in the upper pitcher tube. This transition was characteristic for all *N. gracilis* pitchers (figure 2*a*, negative indices) and absent in *N. rafflesiana* (figure 2*c*, negative indices).

During upward lid displacement in *N. gracilis*, we observed a uniformly distributed decrease of curvature (straightening) throughout the length of the upper dorsal spine and the proximal portion of the lid (figure 2*b*, indices −8 to +2). Equal loading of the lid resulted in significantly larger downward (3.5 ± 0.5 mm) than upward (1.9 ± 0.2 mm, mean ± s.e.m.) displacement (paired *t*-test: *n* = 10, d.f. = 9, *t* = 5.78, *p* < 0.001; electronic supplementary material, figure S2).

## 4. Discussion

The characteristic ‘springboard' action of the *N. gracilis* lid is underpinned by adaptations of the pitcher geometry that facilitate anisotropic deformation. Previous research postulated that a torsional ‘spring' in the pitcher neck is instrumental for the ‘springboard’ trapping mechanism [12]. We show that this spring consists of two distinct parts and extends further into the pitcher body than previously assumed. The very low cross-sectional area of the *N. gracilis* pitcher neck creates a point of weakness for deformation. By contrast, the *N. rafflesiana* neck has a larger cross-sectional area and is reinforced by large peristome flanges with very tough, lignified tissue [13]. In the lower deformation zone of *N. gracilis*, the transition from convex to concave (figure 2*a*) creates a further point of weakness where the rear wall aligns with the axis of bending. Above, the increasingly concave cross-section causes higher bending resistance, explaining the intermediate region of low deformation. By contrast, the *N. rafflesiana* pitcher has an approximately triangular cross-section, with a pronounced angle throughout the rear spine (figure 2*c*). Here, the weakest point is the basal part of the lid, where the tissue is thin and the cross-sectional area is lowest.

Our µ-CT scans show that the axis of bending is off-centre in the dorsal spine of the pitcher tube, rendering the deformation direction-dependent. When the lid moves down and the dorsal spine bends forward, the upper rear section of the pitcher is pushed inward, indenting the open end of the pitcher tube. This is facilitated by the v-shaped profile in this part of the dorsal pitcher wall. When the lid moves up, the tubular shape, reinforced by the peristome at its upper rim, strongly resists the outward extension. The absence of localized deformation zones suggests that this resistance is homogeneous along the dorsal spine. Thus, the *N. gracilis* lid spring is anisotropic, with significantly larger downward than upward displacement under equal loading (electronic supplementary material, figure S2). This may facilitate rapid downward acceleration and high jerk forces at the bottom of the initial downstroke, causing insects to fall into the pitcher [12]. The increased resistance during the upstroke limits the amplitude which may promote damping and speed up the resetting of the trap.

The off-centre axis of bending restricts the possible direction of movement to the pure up–down pivoting that is essential for ‘springboard' trapping [12]. Impact-induced oscillations are not unique to *N. gracilis* lids, but occur in all leaves, where they aid water shedding (reviewed in [14]) and thereby the dispersal of contaminants and pathogen spores [15]. In contrast to the clean pivoting of the *N. gracilis* lid, most leaves show a complex mixture of flapping, bending and twisting [16]. A similar leaf-like impact response is also typical for the lids of *N. rafflesiana* pitchers [12]. It is conceivable that such complex, three-dimensional movement facilitates water shedding as changes in both proximal–distal and lateral leaf inclination angle minimize the distance for surface water to travel in order to drip off.

Our results point to a key role of pitcher geometry for the ‘springboard' function of the *N. gracilis* lid. Geometrical optimization for structural stability and damage resistance is ubiquitous in plants, and small changes in cross-sectional shape can have profound effects [17]. This may be further enhanced by adaptations of the mechanical tissue properties. Plants can easily fine-tune tissue properties by varying turgor pressure or modifying the thickness, structure and chemical composition of their cell walls [18,19]. All vascular bundles have to pass through the narrow constriction of the pitcher neck, where they make up the bulk of the tissue. Reinforced by fibrous sclerenchyma tissue and thickened, lignified cell walls, vascular bundles are typically an order of magnitude stiffer than parenchyma tissue [20]. Future studies should disentangle the effects of pitcher geometry and mechanical tissue properties.

*Nepenthes* species differ in the relative investment in their traps [21], alternative trapping mechanisms and associated morphological components [22,23]. While *N. rafflesiana* pitchers secret most nectar at the inner edge of the peristome, *N. gracilis* allocates a larger proportion to the lower lid surface [5]. Compared with *N. rafflesiana*, *N. gracilis* pitchers have smaller peristomes [23], but higher overall lignin content, pointing to increased structural reinforcement [24]. This might explain why *N. gracilis* pitchers are less prone to turgor loss and temporary drooping of the lid during hot days (U. Bauer 2014, unpublished field observations). More detailed studies of pitcher tissue composition are needed to establish whether structural components are distributed evenly throughout the pitcher or concentrated in certain structures such as the extremely rigid peristome.

In line with the conceptual framework of a ubiquitously applicable leaf economic spectrum [25], the structural reinforcement of *N. gracilis* pitchers is paired with a two to three times longer pitcher lifespan compared with *N. rafflesiana* ([24], and personal observations). Carnivorous plants are thought to reside on the slow-growing, resource-intensive, long-lived end of the leaf economic spectrum [26]. Our study highlights that implications for specialized leaf functions such as the ‘springboard' trapping mechanism may also drive the evolution of mechanical leaf properties, potentially explaining why carnivorous plant species have repeatedly appeared as outliers on the leaf economic spectrum [27,28].

## Data Availability

All data (csv.files, code, images, CT scans) are accessible from the Dryad Data Repository: https://doi.org/10.5061/dryad.7h44j0zw3 [29]. Further method descriptions and analysed data are provided as electronic supplementary material [30].
